# A Cohort Review Approach Evaluating Community Health Worker Programs in New York City, 2015–2017

**DOI:** 10.5888/pcd16.180623

**Published:** 2019-07-11

**Authors:** Alexis Feinberg, Lois Seidl, Rachel Dannefer, Katarzyna Wyka, Elizabeth Drackett, La’Shawn Brown-Dudley, Nadia Islam, Lorna E. Thorpe

**Affiliations:** 1 Department of Population Health, New York University School of Medicine, New York, New York; 2 New York City Department of Health and Mental Hygiene, Long Island City, New York; 3 City University of New York Graduate School of Public Health and Health Policy, New York, New York

## Abstract

The objective of this study was to describe how a cohort review approach was applied as an evaluation framework for a community health worker intervention among adult residents in 5 public housing developments in New York City in 2015–2017. The cohort review approach involved systematically monitoring participants engaged in the Harlem Health Advocacy Partners program during a given time period (“cohort”) to assess individual outcomes and program performance. We monitored participation status (completed, still active, disengaged, on leave, or died) and health outcomes. In this example of a cohort review, levels of enrollment and program disengagement were higher in cohort 1 than in cohort 2. For 6-month health outcomes, the percentage of participants with hypertension who had controlled blood pressure was static in cohort 1 and improved significantly in cohort 2. The percentage of participants with diabetes who self-reported controlled hemoglobin A_1c_ increased significantly in cohort 1 at 6-month follow-up. The cohort approach highlighted important outcome successes and identified workload challenges affecting recruitment and retention.

**Summary**

**What is already known about this topic?**

Findings from community health worker (CHW) interventions targeting chronic disease prevention and management demonstrate inconsistent results, which may be attributable to funding mechanisms. Monitoring tools developed to address resource constraints, such as the cohort review, have not been used previously to evaluate CHW programs.

**What is added by this report?**

We applied a cohort review approach as an evaluation framework for a community-focused CHW intervention in New York City. We assessed program implementation and outcomes during the first 2 years of the program. The cohort approach highlighted 6-month outcome successes related to hypertension and diabetes control and identified workload challenges affecting recruitment and retention.

**What are the implications for public health practice?**

Adapting a cohort monitoring approach can be useful for evaluating the implementation of CHW programs. Such an approach also addresses issues associated with resource constraints and limited program duration.

## Introduction

Although evidence for the effectiveness of community health workers (CHWs) is mounting, reviews of interventions related to chronic disease prevention and management demonstrate inconsistent results (1–3). One key issue is that many CHW programs are funded through grants or operating budgets that are often unpredictable, unstable, and time limited (4). Such funding mechanisms pose unique challenges: with short-term funding, some health outcomes may not emerge within funded evaluation time frames, and positive benefits of programs, including the adoption and maintenance of behavior change, may not have the opportunity to accrue or be sustained. Another problem is inconsistency in how results are reported.

These challenges have affected other public health interventions focused on sustained patient interactions, and monitoring tools developed in response to these challenges can be adapted for CHW program evaluation. For example, the introduction of an annual review process known as a “cohort review” was an important innovation in the monitoring and evaluation of tuberculosis control efforts (5); it involved systematic monitoring of groups of patients beginning treatment within a given period (“cohort”). Structured indicators allowed local and national comparisons, as well as measurement against previous cohorts, to assess improvements in program recruitment, retention, and outcomes.

We adapted cohort review methods to the evaluation of a CHW program. By standardizing participant status definitions and tracking outcome milestones, CHWs and evaluators can develop an analytic framework to better monitor participation status, participant characteristics, and health outcomes. The cohort process also allows for the assessment of trends of program performance indicators that are actionable for decision makers, particularly when comparison groups are unavailable or are no longer supported by funding sources.

## Purpose and Objective

The objective of this study was to describe how the cohort review approach was applied as an evaluation framework for a community-focused CHW intervention, the Harlem Health Advocacy Partners (HHAP) program, in New York City. HHAP is an ongoing municipal project that aims to improve the health of adults residing in 5 public housing developments in East/Central Harlem. Despite rich histories of community organizing, East/Central Harlem has been subject to policies and processes such as redlining, broken windows policing, and “benign neglect” that have contributed to high levels of poverty and poor health outcomes. HHAP was launched to address health and social conditions in the neighborhood, with the aim of closing racial/ethnic gaps in health and social outcomes between public housing residents in East/Central Harlem and other New Yorkers (6,7). We developed and applied the cohort review approach to the health coaching component of HHAP to assess program implementation and outcomes during the first 2 years of the program.

## Intervention Approach

During the first year of HHAP, 224 participants were enrolled from February through August 2015 (cohort 1), and subsequent cohorts followed an annual enrollment cycle. Cohort 2 enrolled 348 participants from September 2015 through August 2016. Concurrent to cohort 1 enrollment, we recruited a 1-year comparison sample of 176 residents from 5 nearby developments, selected on the basis of frequency-matched sociodemographic characteristics and proximity to the intervention developments (8). After cohort 1, comparison groups were not available.

In addition to a residence requirement, eligibility criteria for health coaching and the comparison group included being aged ≥18 and having at least one of 3 self-reported chronic conditions (asthma, diabetes, or hypertension). Participants who reported ever having received a physician diagnosis of asthma, hypertension, or diabetes were defined as adults with these conditions, on the basis of the following question: “Have you ever been told by a doctor, nurse, or other health professional that you have . . . ?” Both cohort 1 and cohort 2 participants were recruited primarily via community outreach conducted by CHWs, who canvassed the grounds of the selected public housing developments and collaborated with community and senior centers in each development to promote the program. The comparison group was recruited from a random sample telephone survey (9). CHWs attempted to deliver core intervention components within 6 to 12 months of enrollment.

The HHAP intervention includes 4 components: 1) health coaching, 2) navigation of the health care system, 3) wellness activities, including peer support and walking groups, and 4) advocacy to build leadership among residents to address community health needs and improve systems and conditions that influence neighborhood health. The health coaching provided by CHWs also included referrals, emergency interventions during acute-risk situations (eg, morbidly high blood pressure readings, mental health crises), and the setting of one or more SMART (specific, measurable, achievable, results-focused, and time-bound) goals. Additional health care navigation support was available through referrals to a partner organization that assists residents in obtaining medical services and ensures they receive the care to which they are entitled. A full description of the HHAP model is available elsewhere (8).

## Evaluation Methods

For cohort 1, CHWs conducted intake assessments as part of participant enrollment (baseline), and an academic research team from the NYU–CUNY Prevention Research Center (PRC) conducted follow-up assessments. The academic research team conducted all comparison group assessments. Cohort 1 and comparison participants received a $20 cash incentive for completing surveys. For cohort 2, CHWs conducted baseline and follow-up assessments. Surveys were conducted at 3 months, 6 months, 9 months, or 12 months after enrollment. Among participants enrolled in cohort 1 and cohort 2, 209 of 224 (93.3%) in cohort 1 and 233 of 348 (67.0%) in cohort 2 completed any follow-up assessment survey. For this analysis, we tabulated data on 6-month follow-up from both years; the response rate was 85.7% (192 of 224) for cohort 1, 92.6% (163 of 176) for the comparison group, and 41.7% (145 of 348) for cohort 2.

We categorized all HHAP participants into mutually exclusive and exhaustive categories of participation in health coaching: completed, enrolled active, disengaged, on leave, or died (Box). CHWs assigned and updated participant status. The NYU-CUNY PRC collected data on health outcomes in the baseline surveys and follow-up surveys. These outcomes were blood pressure control, blood pressure control among participants with hypertension, and self-reported hemoglobin A_1c_ (HbA_1c_) control among participants with diabetes. Blood pressure was the average of 3 measurements taken at each survey point, and we defined control as systolic blood pressure under 140 mm Hg or diastolic blood pressure under 90 mm Hg (10). We dichotomized self-reported status of glycemic control as controlled if a health professional told a participant their diabetes was within goal and as “uncontrolled or don’t know” if they were told it was not within goal or if they were unaware of their status.

**Box. Definition of Each Category of Participation in the Health Coaching Component of the Harlem Health Advocacy Partners Program, New York City, 2015–2017**

**Table:** 

**Status**	**Definition of Status**
Enrolled	Completed intake
Completed	Health coaching completed
Enrolled active	Still active in health coaching and have not yet completed
Disengaged	No longer participating in health coaching. Includes people referred out, people lost to follow-up, people unable to fit health coaching into their schedule, and people who request to stop participating
On leave	Temporarily on leave from the program
Died	Died while enrolled active

Using SAS version 9.4 for all analyses (SAS Institute Inc), we compared the baseline characteristics of cohort 1 with the baseline characteristics of cohort 2 and the comparison group with *t* test for continuous variables and χ^2^ test for categorical variables. For health outcome variables, we tested significance by cohort between enrollment and 6-month post-enrollment by using the McNemar χ^2^ test. We chose this test because it is widely used and easy to interpret.

## Results

A greater percentage of residents participating in HHAP health coaching than in the comparison group were aged 65 or older and self-reported hypertension (Table 1). Most participants were female and either Hispanic or non-Hispanic black, reflecting the population of the public housing developments (9). Participants were demographically similar to one another across cohorts, except that a greater proportion of cohort 2 participants than cohort 1 or comparison group participants were Hispanic.

**Table 1:** Baseline Characteristics of Study Population, Health Coaching Component of the Harlem Health Advocacy Partners Program, 2015–2017^a^

Characteristic	Cohort 1^b^, No. (%) (n = 224)	Comparison Group^c^, No. (%) (n = 176)	Cohort 2^d^, No. (%) (n = 348)	*P* Value^e^
**Age group, y**				
18-44	21 (9.4)	20 (11.4)	41 (11.8)	.04
45-64	104 (46.6)	102 (58.3)	161 (46.4)	
≥65	98 (44.0)	53 (30.3)	145 (41.8)	
**Sex**				
Male	42 (18.8)	35 (19.9)	78 (21.4)	.55
Female	182 (81.3)	141 (80.1)	270 (77.6)	
**Race/ethnicity**				
Hispanic	111 (50.0)	101 (57.4)	170 (60.1)	.02
Non-Hispanic black	105 (47.3)	71 (40.3)	113 (39.9)	
Other	6 (2.7)	4 (2.3)	0 (0.0)	
**Education**				
≤8th grade	49 (22.2)	23 (13.1)	50 (18.9)	.35
Some high school	51 (23.1)	43 (24.6)	72 (27.2)	
High school diploma or GED	72 (32.6)	50 (28.6)	79 (29.8)	
Some college	33 (14.9)	38 (21.7)	50 (18.9)	
College degree or more	16 (7.2)	21 (12.0)	14 (5.3)	
**Disease prevalence^f^**				
Hypertension	197 (88.0)	127 (72.2)	289 (83.1)	<.001
Diabetes	116 (51.8)	74 (42.1)	172 (49.4)	.13
Asthma	88 (39.3)	87 (49.4)	139 (39.9)	.07
Asthma attack in past year	41 (18.5)	36 (20.7)	56 (16.1)	.55
All 3 conditions (hypertension, diabetes, and asthma)	41 (18.3)	19 (10.8)	57 (16.4)	.12
Smoking^g^	50 (22.4)	58 (33.0)	66 (19.9)	.006

a The Harlem Health Advocacy Partners program is an ongoing municipal project that aims to improve the health of adults residing in 5 public housing developments in East/Central Harlem, New York City (8).

b Recruited from February through August 2015.

c Concurrent to cohort 1 enrollment, a 1-year comparison sample of 176 residents was recruited from 5 nearby developments, selected on the basis of frequency-matched sociodemographic characteristics and proximity to the intervention developments.

d Recruited from September 2015 through August 2016.

e *P* value determined by *t* test for continuous variables and χ^2^ test for categorical variables and compares cohort 1 characteristics with characteristics of cohort 2 and comparison group.

f Eligibility criteria for health coaching and the comparison group included being aged ≥18 and having at least 1 of 3 self-reported chronic conditions (asthma, diabetes, or hypertension). Participants who reported ever having received a physician diagnosis of asthma, hypertension, or diabetes were defined as adults with these conditions, on the basis of the following question: “Have you ever been told by a doctor, nurse, or other health professional that you have . . . ?”

g Smoking was dichotomized into an indicator for smoking every day or some days by using the following question: “Do you now smoke cigarettes every day, some days, or not at all?”

Enrollment increased 55.4% from cohort 1 to cohort 2, from 224 to 348 participants. Of the 224 cohort 1 participants, 216 (96.4%) participants were still active in the program after 6 months, 5 (2.2%) had disengaged, 1 (0.4%) was on leave, and 2 (0.9%) had died. Of the 348 participants enrolled in cohort 2, 303 (87.1%) were still active after 6 months, 39 (11.2%) had disengaged, 2 (0.6%) were on leave, and 2 (0.6%) had died.

The percentage of participants with self-reported hypertension in cohort 1 and controlled blood pressure did not change from baseline to 6-month follow-up (58.8% to 60.1%, *P* = .79) (Table 2). Blood pressure control among residents with hypertension in the comparison group may have worsened from baseline to 6-month follow-up (61.0% to 53.3%, *P* = .16). In cohort 2, the percentage of participants with diagnosed hypertension and controlled blood pressure increased significantly, from 57.7% to 73.9% (*P* = .002). The percentage of participants with self-reported diabetes who reported their HbA_1c_ as controlled increased significantly in cohort 1 (50.0% to 64.3%, *P* = .02), whereas self-reported HbA_1c_ control did not improve among comparison group participants (65.7% to 64.2%, *P* = .74). Although the change was not significant, we found improvements in HbA_1c_ control among cohort 2 participants (72.3% to 83.0%, *P* = .20).

**Table 2:** Health Outcome Measures at Enrollment (Baseline) and 6 Months After Baseline Among Participants Who Completed a 6-Month Follow-Up Assessment, Harlem Health Advocacy Partners, 2015–2017^a^

Health Outcome	Cohort 1^b^ (n = 192)		Comparison Group^c^ (n = 163)		Cohort 2^d ^(n = 146)	
	No. (%)	*P* Value^e^	No. (%)	*P* Value^e^	No. (%)	*P* Value^e^
**No. of participants whose blood pressure was monitored** f	174	—	143	—	132	—
Blood pressure was controlled^g^						
At baseline	108 (62.1)	.89	92 (64.3)	.45	83 (62.9)	.003
At 6-month follow-up	107 (61.5)		87 (60.8)		101 (76.5)	
Maintained control	79 (45.4)	—	68 (47.6)	—	74 (56.1)	—
Control improved	28 (16.1)		19 (13.3)		27 (20.5)	
Control declined	29 (16.7)		24 (16.8)		9 (6.8)	
Maintained uncontrolled	38 (21.8)		32 (22.4)		22 (16.7)	
**No. of participants with self-reported hypertension^h^**	169	—	117	—	124	—
No. of participants whose blood pressure was monitored^f^	153	—	105	—	111	—
Blood pressure was controlled						
At baseline	90 (58.8)	.79	64 (61.0)	.16	64 (57.7)	.002
At 6-month follow-up	92 (60.1)		56 (53.3)		82 (73.9)	
Maintained control	64 (41.8)	—	44 (41.9)	—	56 (50.5)	—
Control improved	28 (18.3)		12 (11.4)		26 (23.4)	
Control declined	26 (17.0)		20 (19.1)		8 (7.2)	
Maintained uncontrolled	35 (22.9)		29 (27.6)		21 (18.9)	
**No. of participants with self-reported diabetes^h^**	101	—	70	—	73	—
**Self-reported HbA_1c_, levels among diagnosed diabetes**	98	—	67	—	47	—
HbA_1c_ was controlled						
At baseline	49 (50.0)	.02	44 (65.7)	.74	34 (72.3)	.20
At 6-month follow-up	63 (64.3)		43 (64.2)		39 (83.0)	
Maintained control	37 (37.8)	—	39 (58.2)	—	29 (61.7)	—
Control improved	26 (26.5)		4 (6.0)		10 (21.3)	
Control declined	12 (12.2)		5 (7.5)		5 (10.6)	
Maintained uncontrolled or “don’t know”	23 (23.5)		19 (28.4)		3 (6.4)	

Abbreviations: —, does not apply; HbA_1c_, hemoglobin A_1c_.

a The Harlem Health Advocacy Partners program is an ongoing municipal project that aims to improve the health of adults residing in 5 public housing developments in East/Central Harlem, New York City (8).

b Recruited from February through August 2015.

c Concurrent to cohort 1 enrollment, a 1-year comparison sample of 176 residents was recruited from 5 nearby developments, selected on the basis of frequency-matched sociodemographic characteristics and proximity to the intervention developments.

d Recruited from September 2015 through August 2016.

e Difference between values at intake and 6-month follow-up examined by using McNemar χ^2^ test.

f Blood pressure measurements were not obtained for every participant because of equipment malfunction, technical errors, or participant refusal.

g Defined as systolic blood pressure <140 mm Hg or diastolic blood pressure <90 mm Hg.

h Participants who reported ever having received a physician diagnosis of hypertension or diabetes were defined as adults with these conditions, on the basis of the following question: “Have you ever been told by a doctor, nurse or other health professional that you have . . . ?”

## Implications for Public Health

Our findings from the first 2 years of HHAP’s health coaching component demonstrate the utility of the cohort review approach in providing a structure for evaluating a multiyear program, particularly when an ongoing comparison group is not available. The approach highlighted successes in health outcomes among participants retained in the program and challenges in program retention.

The assessment showed that more participants in cohort 2 than in cohort 1 disengaged from the program after 6 months. One reason for the higher level of disengagement in cohort 2 could be the challenge of maintaining health-coaching participants carried over from cohort 1 while recruiting for cohort 2, since CHWs in cohort 2 were also responsible for managing participants from the previous year. In addition, in the beginning of cohort 2, programmatic operations were transferred from an external organization to the New York City Department of Health and Mental Hygiene, which may have resulted in a disruption for some participants. Finally, the incentive offered in cohort 1 may have positively influenced program retention and the number of follow-up interviews. Because the cohort process cycle emphasizes continuous monitoring and improvement, the HHAP program addressed retention and workload issues in cohort 3.

Our cohort assessment quantified improvements in key health outcomes shown in previous studies, namely in blood pressure (11) and glycemic control (2,12). The increase from cohort 1 to cohort 2 in the number of participants with controlled blood pressure suggests that the ability of CHWs to enhance care increases over time. This care includes efforts to keep participants connected with their primary care physician and to motivate participants to take all routine tests and medications for their conditions.

In planning for evaluating CHW programs using a cohort approach, metrics for the implementation process should be developed a priori and aligned with program objectives. Our analysis underscored the challenge of defining the participation status of a participant as complete. The definition was challenging because the criteria for program completion changed over 2 cohort years; awareness of this challenge helped formalize the definition of completion. Moreover, the program further disaggregated the disengaged group into 4 new categories: withdrew, lost to follow-up, transferred out of health coaching, and unavailable (ie, unable to fit health coaching into their schedule). It will be important to monitor these categories to assess whether participants are not interested or able to participate in the program, which would reflect a poor fit between the program and a participant’s needs (withdrew), or the program is unable to maintain contact with participants because of other factors (lost to follow-up).

We found the cohort review approach adaptable to new program goals. For example, to better HHAP’s efforts to address the social determinants of health in addition to disease management, we developed outcome metrics for social determinants of health for cohort 3, and the program will continue to monitor other variables that may contribute to health outcomes.

Our study has several limitations. Our findings in part reflect differences in HHAP programmatic operations between cohort 1 and 2. Cohort 1 data were collected by both CHWs and an academic research team and participants in cohort 1 received a cash incentive for completing surveys, whereas cohort 2 data were collected by CHWs only, often with fewer follow-up assessments, and cohort 2 participants did not receive an incentive. Differences in data collection may have biased comparisons between cohort 1 and cohort 2. Some health outcome data were self-reported; however, any bias introduced by self-report is unlikely to be differential across cohorts, except if selection bias was introduced because of higher loss to follow-up in cohort 2. Finally, given the large number of disengaged participants in cohort 2, we are not fully confident that improved outcomes were solely a function of programmatic improvements. Improved outcomes may reflect differential disengagement of participants who would have been less likely to improve.

Although previous CHW evaluations focused on individual-level outcomes, we found the cohort monitoring approach to be an effective method for evaluating the implementation process of CHW programs while also addressing issues associated with resource constraints and limited program duration (13). Adapting a cohort approach can begin to fill this gap (4,14).
